# New active site oriented glyoxyl-agarose derivatives of *Escherichia coli *penicillin G acylase

**DOI:** 10.1186/1472-6750-7-54

**Published:** 2007-09-10

**Authors:** Davide A Cecchini, Immacolata Serra, Daniela Ubiali, Marco Terreni, Alessandra M Albertini

**Affiliations:** 1 Dipartimento di Genetica e Microbiologia, via Ferrata 1, Università degli Studi di Pavia, 27100 Pavia, Italy; 2 Dipartimento di Chimica Farmaceutica, Pharmaceutical Biocatalysis Laboratories, via Taramelli 12, Università degli Studi di Pavia, 27100 Pavia, Italy

## Abstract

**Background:**

Immobilized Penicillin G Acylase (PGA) derivatives are biocatalysts that are industrially used for the hydrolysis of Penicillin G by fermentation and for the kinetically controlled synthesis of semi-synthetic β-lactam antibiotics. One of the most used supports for immobilization is glyoxyl-activated agarose, which binds the protein by reacting through its superficial Lys residues. Since in *E. coli *PGA Lys are also present near the active site, an immobilization that occurs through these residues may negatively affect the performance of the biocatalyst due to the difficult diffusion of the substrate into the active site. A preferential orientation of the enzyme with the active site far from the support surface would be desirable to avoid this problem.

**Results:**

Here we report how it is possible to induce a preferential orientation of the protein during the binding process on aldehyde activated supports. A superficial region of PGA, which is located on the opposite side of the active site, is enriched in its Lys content. The binding of the enzyme onto the support is consequently forced through the Lys rich region, thus leaving the active site fully accessible to the substrate. Different mutants with an increasing number of Lys have been designed and, when active, immobilized onto glyoxyl agarose. The synthetic performances of these new catalysts were compared with those of the immobilized wild-type (wt) PGA. Our results show that, while the synthetic performance of the wt PGA sensitively decreases after immobilization, the Lys enriched mutants have similar performances to the free enzyme even after immobilization.

We also report the observations made with other mutants which were unable to undergo a successful maturation process for the production of active enzymes or which resulted toxic for the host cell.

**Conclusion:**

The desired orientation of immobilized PGA with the active site freely accessible can be obtained by increasing the density of Lys residues on a predetermined region of the enzyme. The newly designed biocatalysts display improved synthetic performances and are able to maintain a similar activity to the free enzymes. Finally, we found that the activity of the immobilized enzyme proportionally improves with the number of introduced Lys.

## Background

Penicillin G acylases (PGA, also known as PA, benzylpenicillin acylase, penicillin amidase, penamidase or acyl transferase), are enzymes widely distributed among micro-organisms in the living world. The physiological role of these enzymes is still unclear, but in *Escherichia coli *the PGA expression is regulated both by temperature and by phenylacetic acid, suggesting that the enzyme could be involved *in vivo *in the assimilation of aromatic compounds to be used as carbon source [1]. In *E. coli*, PGA is a periplasmic enzyme organized as a αβ heterodimer. The two subunits, α (24 kDa, 209 residues) and β (65 kDa, 566 residues), are produced through a complex maturation process of a pre-pro-PGA polypeptide precursor of 95 kDa: at the N-terminus, the signal peptide that directs the export of the enzyme, is removed to give pro-PGA; this step is followed by an auto-proteolytic cleavage of a 54 residues spacer peptide [2,3].

It is believed that an appropriate folding status of pre-pro-PGA and pro-PGA should be obtained for subsequent processing, and that such a folding process may possibly be assisted by chaperons. During the steps of gene expression (transcription, translation and post-traslation modification), a balanced protein synthesis flux should be properly maintained in order to avoid the accumulation of any protein species within the protein formation pathway [4-6].

During the overproduction of the enzyme, the final yield is limited by misfolding of pre-pro-PGA and pro-PGA, which results in the aggregation of insoluble precursors as inclusion bodies and in an inefficient maturation that consequently limits the formation of active PGA. Xu et al. [4,5] demonstrated that the cytoplasmic overexpression, the folding and the processing of PGA can be improved by co-expressing PGA with cytoplasmic chaperones such as Trigger factor, GroEL/ES and DnaK/J-GrpE. Their results suggest that the chaperone team of Trigger factor and GroEL/ES collaboratively facilitated the proper folding of leaderless pro-PGA, by protecting pro-PGA from proteolysis, which improved the pro-PGA maturation. The same results were obtained by the co-expression of periplasmic DegP chaperone [6].

Structural characterization of mature PGA and of its precursor has elucidated the catalytic properties and the processes of maturation [7-14].

PGA from *E. coli *ATCC 11105, EC 3.5.1.11 is a commercially valuable enzyme which is used as a catalyst for the production of β-lactam nuclei, starting from penicillin G (obtained by fermentation) and semi-synthetic cephalosporin G, and for the kinetically controlled synthesis of several semi-synthetic antibiotics [15-22]. The expression of the *pac *gene for PGA in native and recombinant *E. coli *has been studied and improved to obtain a higher production of the enzyme [21,22].

The recovery and reuse of PGA as the catalyst in synthetic applications is routinely performed by using the immobilized enzyme on a solid support by covalent interaction [23]; alternatively, reversible immobilization on ionic exchangers, which is improved by site directed mutagenesis, offers simpler and inexpensive immobilization protocols [24].

Other advantages of immobilized enzymes are the enhanced stability of the resulting derivatives and the possibility to modulate their catalytic properties [25]. These properties may be affected by the orientation of the enzyme as well as by the microenvironment of the support around the active site. These effects become particularly important if the enzyme is immobilized with the active site near or facing the surface of the support. Many efforts have been made to control the orientation of immobilized enzymes of commercial use such as subtilisin, for example by binding it through a predetermined site of the protein surface [26].

Several insoluble supports can be used to exploit different functional groups that are distributed on the enzyme surface for immobilization. Some of the most studied and employed supports are glyoxyl activated supports, in which the immobilization proceeds by interacting with the richest Lys area of the protein surface [27].

In PGA, Lys residues are uniformly distributed on the enzyme surface, and a few are also present near the active site [11,12]: if the binding to the solid support occurs through one of the latter, PGA may undergo a structural distortion which can hamper the accessibility of the substrates into the active site that leads to a partial loss of activity. The increase in Lys content far from the active site has been shown to be a suitable approach to increase the number of bonds between the mutated PGA and the glyoxyl-support, leading to a more stable derivative [28].

This result seems to indicate that the immobilization of this PGA mutant preferentially occurs through the enzyme surface which is enriched with the three "extra" Lys residues; consequently, the active center is oriented toward the opposite side of the support surface. The "preferential" arrangement of the enzyme should reduce any diffusion problem as glyoxyl-derivatives make the active site more accessible to the β-lactam nuclei. In fact, similar results were obtained when a 3 Gly -3 Lys tag was added at the C-terminus of the β subunit [29].

**Figure 1:**
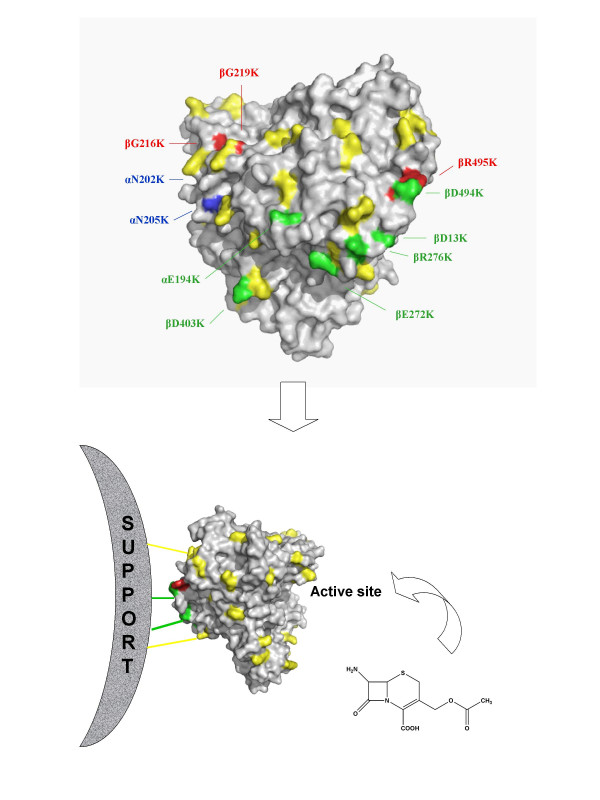
Surface model of the three-dimensional structure of the rear-face of the mutated PGA. The existing Lys in the wt protein are yellow; the residues introduced by PCR mutagenesis that resulted in an active enzyme are in green; residues that had a negative effect are in blue and red, which indicate toxicity or maturation defect, respectively (top of the figure). The desired effect is to create the link with the resin on the rear-face of the protein in order to obtain a derivative with a freely accessible active site for the substrate (bottom of the figure).

To verify this hypothesis, we designed different aminoacid substitutions on the enzyme surface which is opposite to the active site, and we tested some of these immobilized derivatives along with the three Lys enriched enzyme derivative [28] in kinetically controlled acylation of 7-ACA. The acylation assay should highlight the difference in the access of the nucleus between the immobilized mutant and the immobilized wt PGA (Figure 1). The newly mutated enzymes, together with other mutated *pac *gene products, which were obtained by site directed mutagenesis of the *E. coli *ATCC 11105 gene, have been evaluated for their expression and maturation in recombinant *E. coli *and for their activity in solution and after immobilization.

## Results and discussion

### Site directed mutagenesis

The crystal structure of the native PGA clearly shows that Lys residues are uniformly distributed on the surface of the enzyme [8,11]. Aminoacid substitutions of flexible superficial residues were designed in order to increase the number of Lys in an opposite region to the active center. Site directed mutagenesis reactions of the *pac *gene were performed on plasmid pETPAC [29] by using mutagenic overlapping primers extension for the PCR reaction (QuickChange IIXL Stratagene). Single, non-conservative substitutions that replace acidic and basic residues (αE194K, βD403K βD494K, βR495K and βD216K) were obtained by using the primers that are shown in Table 1 and by using the pETPAC plasmid. The new pETPAC-E194K plasmid was then used as a template to obtain the double mutant (αE194K-βD494K). The other mutants with two substitutions, (βD216K-βG219K) and (αN202K-αN205K), were created by single step mutagenesis. The triple mutant PGA-3K was obtained from the pOAF3mut plasmid [28]. The mutants with four and five substitutions were obtained by using the pOAF3mut plasmid as a template and the oligonucleotides D494K for and D494Krev, R495Kfor and R495Krev, and D216K-G219K. All the mutations were confirmed by DNA sequencing. The residues substituted by Lys in the structure of the mature PGA are depicted in Figure 1.

**Table 1:** Forward and reverse primers used for the PCR mutagenesis

**Name**	Sequence
**D216K for**	5'-GGTACGGGAAAATGG***A***A***G***TGGAAAGGGCTATTGCCTTTTG-3'
**D216K rev**	5'-CAAAAGGCAATAGCCCTTTCCA***C***T***T***CCATTTTCCCGTACC-3'
**E194K for**	5'-CCACTATTGCCGTACAA***A***AGAGTAACTACCCAC-3'
**E194K rev**	5'-GTGGGTAGTTACTCT***T***TTGTACGGCAATAGTGG-3'
**D403K for**	5'-AGGCGGTGCAGGGA***A***A***G***AAATCACCAATCCC-3'
**D403K rev**	5'-GGGATTGGTGAT***T***T***C***TTTCCCTGCACCGCCT-3'
**D494K for**	5'-CCAACGACAAGC***A***A***A***CGTCCTGTGCTTGCC-3'
**D494K rev**	5'-GGCAAGCACAGGACG***T***T***T***GCTTGTCGTTGG-3'
**R495K for**	5'-CAACGACAAGCGAT***AAG***CCTGTGCTTGCC-3'
**R495K rev**	5'-GGCAAGCACAGG***CTT***ATCGCTTGTCGTTGG-3'
**D216K/G219K for**	5'-ACGGGAAAATGG***A***A***G***TGGAAA***AA***GCTATTGCCT-3'
**D216K/G219K rev**	5'-AGGCAATAGC***TT***TTTCCA***C***T***T***CCATTTTCCCGT-3'
**N202K/N205K for**	5'-CCCACTTAAATTTAA***G***CAGCAAAA***G***TCGCAAACAGC-3'
**N202K/N205K rev**	5'-GCTGTTTGCGA***C***TTTTGCTG***C***TTAAATTTAAGTGGG-3'

### Enzyme screening and assay

100 ml cultures of *E. coli *BL21(DE3) cells, which were transformed with the corresponding plasmid, were used to overexpress the mutated and native enzymes. Total cellular proteins were analysed by SDS-PAGE. The results are summarized in Table 2: with the exception of the double mutant PGA-αN202K/αN205K, all the recombinant proteins were overexpressed, but only PGA-αE194K, PGA-D403K, PGA-βD494K, the double PGA-αE194K-βD494K mutant and the triple, PGA-βD13K-E272K-R276K [28] mutant enzymes, also named herein 2K and 3K respectively, were found in the periplasmic extract in the processed and mature α and β subunits form. The presence of the other mutant acylases in soluble periplasmic or cytoplasmic extracts was negligible; they were mainly localized in the pellet as inclusion bodies of pre-pro-enzyme precursor. The double substitution of residues N202 and N205 of the α subunit dramatically affected the expression of the acylase. No enzyme was observed in the periplasmic, cytoplasmic, and pellet fractions; the presence of rearranged pET-PAC-αN202K-αN205K expression vectors indicated a possible selection against the production of a toxic protein.

**Table 2:** Activities of the new mutants obtained by single or multiple combinations of aminoacid substitutions

**N. of Lys introduced**	**Mutants**	**Expression of the 95 kDa pre-pro-enzyme**	**Enzyme processing^a^**	**U_(NIPAB)_/ml^b^**	**U_(NIPAB)_/mg**
0	Wt	+	+	25.0	5.0
1	αE194K	+	+	23.2	5.3
	βD494K	+	+	27.0	5.4
	βD403K	+	+	13.9	4.6
	βR495K	+	-	n.d.^c^	n.d.^c^
	βD216K	+	-	n.d.^c^	n.d.^c^
2	αE194K, βD494K	+	+	20.0	4.0
	βD216K, βG219K	+	-	n.d.^c^	n.d.^c^
	αN202K, αN205K	-	-	n.d.^c^	n.d.^c^
3	βD13K, βE272K, βR276K (3K)	+	+	19.0	4.3
4	(3K)+ βD494K	+	-	n.d.^c^	n.d^c^.
	(3K)+ βR495K	+	-	n.d.^c^	n.d.^c^
5	3K+ βD216K+βG219K	+	-	n.d.^c^	n.d.^c^

^a^Enzyme processing means presence of the two 65 and 24 kDa subunits in the SDS-PAGE analysis of periplasmic extract.

^b^U, units are μmol of NIPAB hydrolyzed min^-1 ^ml^-1 ^of periplasmic extract.

^c^n.d., means not detectable enzyme activity.

As previously observed [9,11,13,30], overexpression, processing and maturation can be severely affected by temperature and this drawback can be enhanced by the presence of single residue mutations. We used 22°C for the induction which has been reported to be the ideal temperature also in the case of a mutated Gly-Lys tagged PGA [29]. Other substitutions βS1C, βS1T, βS1R, βS1G, H26G, Q118E, S9E, V97K [2,31] have been previously demonstrated to be detrimental for the correct maturation and activity of PGA. Our data indicate that the two surface residues Asn 202 and 205 in α, and the two Arg 495 and Asp 216 in the β subunit cannot be substituted by Lys without destabilizing the enzyme structure (as suggested by the presence of insoluble aggregates or by the absence of any precursor or mature PGA): in the case of the latter two substitutions, the precursor proteins of 95 kDa are expressed but not translocated and cleaved in the periplasm. The additional substitution of Asp 494 with Lys in the β chain, which alone leaves the autocatalytic transport and localization properties unimpaired, also affected the stability when the three mutations D13K-E272K-R276K were present in the same chain. Whereas, substitution of Asn 202 and Asn 205 at the C-terminus of the α subunit seems to be more dramatic. In this case, the overexpression of the unprocessed enzyme was affected, probably because the substituted residues which are located near one of the auto-proteolytic sites, i.e. the A209-A210 peptide bond which is hydrolyzed to eliminate the spacer peptide during the maturation process, interferes with the correct maturation process by producing a by-product polypeptide highly toxic for the host cell.

The hydrolytic activity of periplasmic extract of all the mutant acylases was evaluated by using the NIPAB assay [14]. Activity (expressed as μmol min^-1 ^mg^-1^of NIPAB hydrolyzed, Table 2) was found only in the periplasmic extract of the acylases whose processing was verified by SDS-PAGE electrophoresis analysis (data not shown). In the periplasmic extract of the other recombinant acylases, the activity was undetectable: neither the cytoplasmic extract, nor the pellet, which was resuspended in the same buffer, showed the presence of hydrolytic activity. The absence of activity, and the fact that the acylases were found mainly in the pellet as non-mature enzymes suggest that the aminoacid substitutions were incompatible with a successful maturation, probably as a consequence of an incorrect folding that leads to the formation of non-soluble inclusion bodies.

### Enzyme purification and characterization

**Figure 2:**
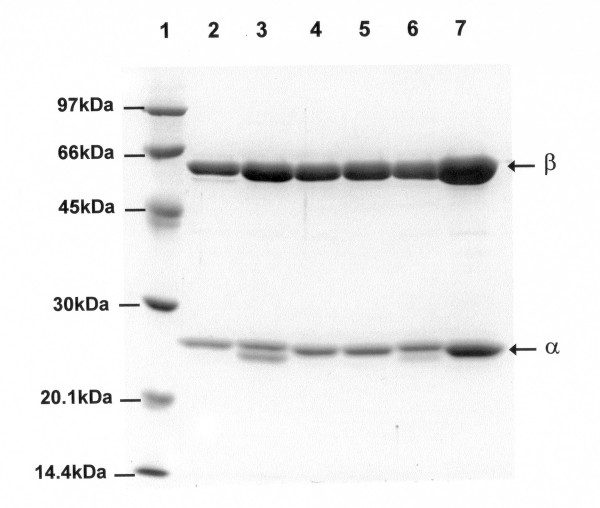
SDS-PAGE analysis of the purified native and mutated acylases. Arrows indicate the α (24 KDa) and β subunit (65 Kda) of the mature enzymes. Lane 1: MW marker; Lane 2: PGA wt; Lane 3: PGA-αE194K; Lane 4: PGA-βD494K; Lane 5: PGA-βD403K; Lane 6: PGA-2K (PGA-αE194K-βD494K); Lane 7: PGA-3K (PGA-βD13K-E272K-R276K).

The five acylases that resulted active and the wt enzyme were overproduced from scaled-up 1L cultures and purified to be subsequently immobilized. The purified native and recombinant enzymes (purity >90%, Figure 2) were assayed for the hydrolytic activity by using both NIPAB [14] and penicillin G potassium salt (PGK) as substrates (see Methods). All the recombinant acylases in solution showed hydrolytic activities comparable to the one of the native enzyme (Table 3). The presence of a secondary band of less than 24 kDa in the two PGA-αE194K and PGA-αE194K-βD494K purified enzymes could be explained by the presence of a contaminant peptide or a maturation by-product, both, in some way, dependent on the presence of the αE194K substitution. In order to define this aspect, the N-terminal sequence of this secondary band was determined by automated sequencing, which confirmed the sequence of the α subunit and suggested a C-terminus processing that reduces the polypeptide size.

**Table 3:** Specific activities of the purified acylases

**Purified enzyme**	**U^a^_NIPAB_/mg**	**U^a^_PGK_/mg**
**PGA wt**	18.5	54.0
**PGA-αE194K**	14.0	40.0
**PGA-βD494K**	21.0	46.6
**PGA-βD403K**	12.8	46.3
**PGA-2K**	16.4	45.0
**PGA-3K**	14.0	25.0

^a^U, μmol of substrate hydrolyzed min^-1 ^mg^-1^.

### Enzymes immobilization and determination of the activity

**Figure 3:**
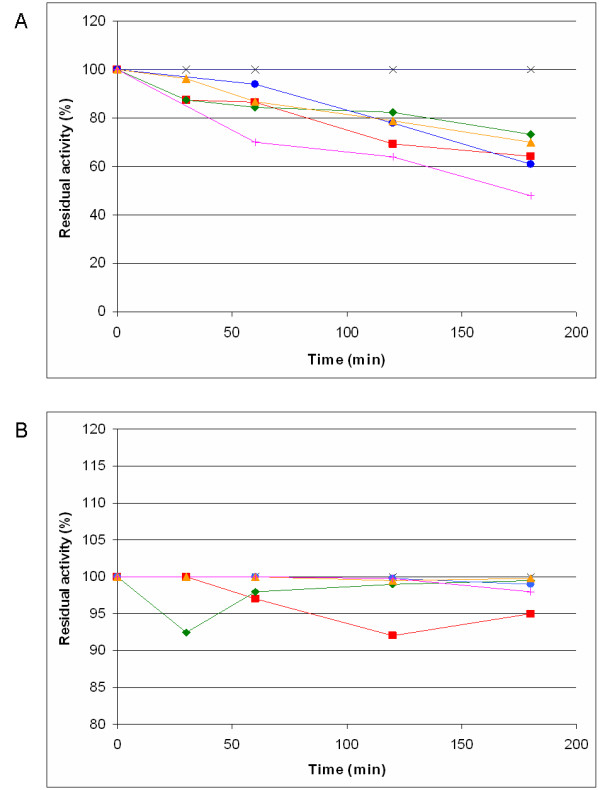
Stability of the mutated acylases compared to the wt enzyme. Residual activity at pH 10, at 25°C in A and at 4°C in B. Symbols: × (black), PGA wt; ■ (red), PGA-αE194K; ◆ (green), PGA-βD494K; ● (blue), PGA-βD403K; ▲ (amber), PGA-2K; + (pink), PGA-3K.

The native and modified PGAs were immobilized on aldehyde agarose, i.e. agarose activated with glyoxyl groups. Since the extreme alkaline conditions (pH 10) which are necessary to obtain the optimal yield of immobilization [26,29,32,33] may affect the stability of the mutated enzyme, a preliminary stability study was performed. The results are shown in Figure 3: at pH 10 and 25°C, all the mutated acylases were unstable and lost up to ~50% activity in less than three hours, but the activity was retained up to 100% simply by lowering the temperature to 4°C. In these conditions, the amount of immobilized protein was quantifiable for all the acylases (95–100%, Table 4). After immobilization, the PGK activities of all the acylases decreased, but the mutants were able to retain more than 50% of the activity, while the native enzyme usually loses up to 50% of its activity.

**Table 4:** Immobilization of acylases

**Enzymea**	**U_PGK_^b^loaded**	**Protein loaded (mg/g of support)**	**% of protein immobilized**	**Activity (U_PGK_^b^/g of support)**	**Specific activity (U_PGK_/mg of immobilized enzyme)^c^**
**PGA wt**	100	1.8	97	45.2	26.5 ± (0.5)
**PGA-αE194K**	100	2.8	100	63.4	22.0 ± (0.7)
**PGA-βD494K**	138	3.0	95	73.2	24.4 ± (0.4)
**PGA-βD403K**	100	2.2	97	54.6	26.0 ± (0.6)
**PGA-2K**	139	3.0	97	86.2	28.7 ± (0.8)
**PGA-3K**	80	3.2	97	54.0	17.4 ± (0.2)

^a^The enzymes were immobilized on agarose beads activated with aldehyde groups at pH 10 and 4°C.

^b^U, μmol of PGK min^-1 ^mg^-1^.

^c^Activities of immobilized enzymes are expressed as units (μmol of PGK hydrolyzed min^-1 ^mg^-1^) per gram of agarose beads.

### Determination of the vs/vh_1 _ratio

The soluble enzymes and their immobilized derivatives were tested as catalysts in the kinetically controlled N-acylation of 5 mM 7-aminocephalosporanic acid (7-ACA) in the presence of 5 mM R-mandelic acid methyl ester (MAME), by evaluating the ratio between the initial rate of synthesis (vs) and the ester hydrolysis (vh_1_) [25].

**Figure 4:**
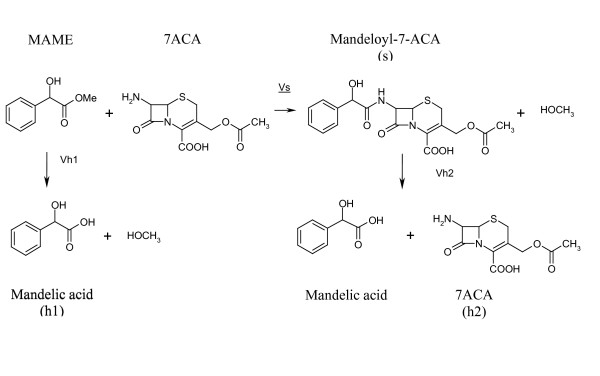
Reactions involved in the kinetically controlled synthesis of β-lactam antibiotics.

In the kinetically controlled synthesis, the yields depend on the balance of three different reactions shown in Figure 4. These reactions are the synthesis of the β-lactam compound (s), the hydrolysis of the activated acyl donor (h_1_) and the hydrolysis of the synthesized antibiotic (h_2_). The higher achieved yields are transient and are dependent on the saturation degree and on the affinity of the enzyme active center for the nucleophile. In particular, the ratio between the rate of synthesis and the ester hydrolysis (vs/vh_1_) is strictly dependent on the affinity of the enzyme for the β-lactam nucleus and is a parameter for the ability of the catalyst to adsorb the substrate into the active center.

**Figure 5:**
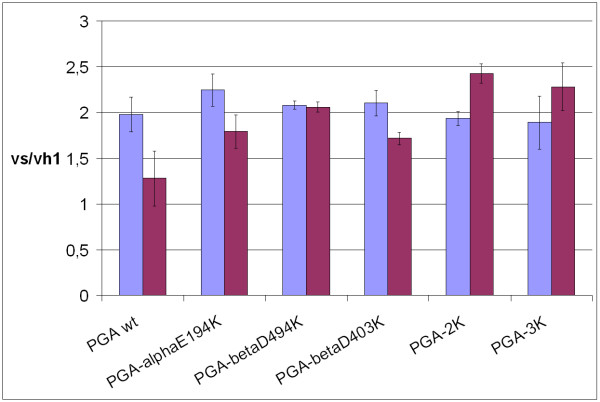
vs/vh_1 _ratio in the acylation reaction of β-lactam nuclei. vs/vh_1 _ratio for the N-acylation of 7-ACA with MAME was evaluated at pH 6.5 and 4°C (5 mM). The ratio of all the glyoxyl agarose (violet column) derivatives, which were obtained with the native and mutated acylases, was compared to that of the soluble enzymes (light blue column).

The results are shown in Figure 5: all of the soluble mutant PGAs exhibited a vs/vh_1 _ratio similar to the one of the native enzyme (~2). Thus, the effect of the aminoacid substitutions on the catalytic properties is negligible.

On the other hand, after immobilization the mutant PGAs showed higher vs/vh_1 _ratios than the wt, which seem to increase proportionally with the number of Lys introduced: from a value of 1.3, for the native enzyme, to 2.3, for the PGA3K, and of 2.5 for PGA2K. These data, if compared to the results that were obtained with the soluble acylases, indicate that after immobilization the recombinant enzymes allow a better diffusion and adsorption of the β-lactam nucleus into the active site than the one observed with the wt enzyme.

We propose the following hypothesis to explain our observations: the enrichment of Lys on the enzyme surface enhances the immobilization of a higher amount of PGA molecules with a "preferential" orientation, which leaves the corresponding active centers exposed to the reaction environment and more easily accessible to the β-lactam nucleus.

## Conclusion

The present results demonstrate that glyoxyl supports are suitable to induce a preferential orientation during immobilization of PGA mutants with an increased number of Lys in a specific area of the protein surface. These results are in agreement with those previously reported [28,29].

This effect has not been obtained with other methods of immobilization such as ionic or covalent interactions based on a different binding chemistry [24,35].

We have designed and obtained four new acylases which are characterized by an increased content of Lys on the surface of the protein, which is localized on the opposite side of the active center. We compared the performances of these new immobilized and mutated PGAs with those of the three Lys mutants 3K that were described by Abian et al. [28].

The kinetically controlled reactions performed by the immobilized mutants and the immobilized wild-type PGA showed that the increasing number of Lys residues on the surface that is localized on the opposite side of the active site, positively correlates with a proportionally higher diffusion of the β-lactam nucleus in the catalytic site.

This effect was particularly evident when two or three Lys were introduced by mutagenesis. The obtained results seem to indicate that these substitutions enhance the immobilization of PGA through the Lys enriched part of the surface, and allow the immobilized catalysts to expose the active site to the reaction environment (Figure 1).

Although these substitutions have been found to decrease the specific hydrolytic activity of the soluble enzymes, if compared with the native enzyme, they had negligible effects on the vs/vh1 ratio. When assayed in the kinetically controlled acylation of 7-ACA, the glyoxyl-agarose derivatives with two and three Lys showed a higher vs/vh1 ratio, than the soluble enzymes (Figure 4). These results can be explained if we suppose that the enzyme immobilization was driven by the Lys-enriched surface, which exposes the enzyme active site to the reaction environment, therefore easily accessible for the substrate. We are setting up MALDI-TOF analysis of peptides obtained by tryptic digestion of the enzyme which is immobilized on glyoxyl-agarose and epoxydic resins, derivatives which involve a different binding chemistry. This approach can directly demonstrate the effective role of the mutations in the enzyme orientation.

We have also obtained six other new mutant *pac *genes which resulted inactive in NIPAB assay in crude extracts when overexpressed, and which showed a SDS-PAGE pattern typical of misfolded and aggregated expression products. These proteins were found as precipitated inclusion bodies that were formed by unprocessed precursor. These data demonstrate an enhancement of the previously observed behaviour for the wild-type enzyme when overexpressed [4-6].

We think that the Lys substitution of residues of the β subunit (single mutants βD216K, βR495K; double mutants βD216K-G219K and all four and five Lys derivatives of the 3K mutants, Table 2), severely impaired the enzyme folding and the stability in an overexpression system that has been demonstrated to be crucial even for the wild-type PGA [4-6], because it blocks the following autocatalytic steps of enzyme maturation and periplasmic translocation. The same effect was observed when Asp 494 was substituted by Lys in the three Lys enriched mutant of Abian et al. [28].

These substitutions are located far from the autocatalytic site of the precursor and on the opposite side of the precursor structure, in the same way that all the other mutations we planned [2,12].

It is noteworthy that, in three cases the simple addition of a Lys residue near the substitutions that were compatible with the maturation and the activity, leads to the impairment of the enzyme maturation.

The double substitution of Asn residues 202 and 205 with Lys, at the C-terminus of the α subunit, gave rise to the more dramatic effect of the total absence of an overexpressed protein, even as unfolded aggregate in the pellet fraction. This dramatic effect was probably due to a higher toxicity of the mutant versions of the enzyme precursor in the producer host and to its consequent negative selection.

We could overcome the problems found in the expression of these mutants, and finally test their performances as immobilized derivatives, by using the engineered strains and the two-step protocols described by de Marco et al. [36] that recently have been demonstrated to strongly enhance the solubility of several overexpressed proteins.

## Methods

### Chemicals

Penicillin G potassium salt (PGK) and 6-nitro-3-phenylacetamido benzoic acid (NIPAB) were purchased from SIGMA-Aldrich (Milano, Italy). R-mandelic acid methyl ester (MAME) was purchased from Fluka (Milano, Italy). 7-Amino-cephalosporanic acid (7-ACA) was purchased from Farmabios (Gropello Cairoli, Italy). Agarose CL6B was purchased from Pharmacia Biotech AB (Uppsala, Sweden). All solvents were of HPLC grade and purchased from SIGMA-Aldrich.

### Mutagenesis

The site directed mutagenesis was made by using the method of mutagenic overlapping primers extension by *PfuUltra *made PCR (QuickChange IIXL Stratagene) on the plasmids pET-PAC [29], pOAF3mut [28] (gift of O. Abian) and on the derivatives pETPAC-E194K and pETPAC-D216K, by using the forward and reverse primers listed in Table 1.

### Enzyme overexpression

Culture of *E. coli *BL21(DE3) of 100 ml Luria-Bertani medium supplemented with 100 μg/ml Ampicillin were used to evaluate the overexpression of the mutated enzymes. Cells were grown at 28°C until the OD_600 _reached 0.6–0.8. Then, the cultures were induced with 0.5 mM IPTG (isopropyl-β-D-thiogalactopyranoside) and the temperature was lowered to 22°C to balance yield, processing and maturation [9,10,26,27]. After 24 hours from induction, cells were harvested by centrifugation at 5000 rpm for 30 min and the periplasmic proteins were extracted by osmotic shock at 4°C [7]. To evaluate the proteins which were expressed as precursor in inclusion bodies, after centrifugation at 13000 rpm for 30 min, the spheroplasts were re-suspended in 50 mM Tris-HCl pH 8.5 and disrupted by sonication. Cell debris was harvested by centrifugation at 13000 rpm for 10 minutes at 4°C. Total cellular proteins in the crude extract supernatant and pellet were quantified [34] and analysed by SDS-PAGE and Coomassie-Blue staining.

### Enzyme purification

The periplasmic extracts of the active PGA, which were obtained from cultures of 1L as previously described, were brought to pH 8.5 with 50 mM Tris-HCl and loaded on a Q-Sepharose HP 26/10 column (Pharmacia), which was previously equilibrated in the same buffer, and eluted with a linear gradient of 0 to 100 mM NaCl. Fractions with eluted proteins were assayed by SDS-PAGE and those containing the mature PGA were collected and dialyzed against 50 mM K-phosphate buffer pH 7.5 for further assays.

### Enzymes activity assays

Crude extract and purified enzymes were spectrophotometrically assayed at 405 nm by using NIPAB (6-nitro-3-phenylacetamido benzoic acid) 0.15 mM in 10 mM K phosphate buffer pH 7 at 25°C.

One unit of PGA activity was defined as the amount of enzyme that hydrolyzes 1 μmol of NIPAB per minute at pH 7 and 25 °C to give 3-amino-6-nitrobenzoic acid.

Purified enzymes and enzyme derivatives were assayed by automatic titration (718 STAT Titrino Metrohm) with 0.1 M NaOH, of released phenylacetic acid, which is produced by the hydrolysis of 3% Penicillin G potassium salt (PGK) in K phosphate 10 mM at pH 8 and 37°C.

One unit of PGA activity was defined as the amount of enzyme that hydrolyzes 1 μmol of penicillin G per minute at pH 8 and 37°C.

### Preparation of glyoxyl-agarose

Glyoxyl-agarose was prepared as previously reported [35].

### Immobilization

One gram of glyoxyl-agarose was added to 14.3 ml of protein solution prepared in 50 mM NaHCO_3 _and 100 mM phenylacetic acid buffer pH 10. The mixture was gently stirred at 4°C. Samples of the supernatant were periodically withdrawn and the protein concentration was determined by Bradford assay [34]. Finally, the Schiff-base formed between the aldehyde group of the support and the Lys residues were reduced by adding 14 mg of NaBH_4 _under stirring.

### Determination of vs/vh1 ratio

The vs/vh_1 _ratios in the PGA catalyzed N-acylation of 7-aminocephalosporanic acid (7-ACA) with R-mandelic acid methyl ester (MAME) to give mandeloyl-7-ACA (Figure 4) were evaluated by measuring the initial rate of synthesis (vs) and ester hydrolysis (vh_1_). The reactions were performed at pH 6.5 and 4°C. The reactions were carried out in 20 ml phosphate buffer 10 mM; the ester and 7-ACA were dissolved at a concentration of 5 mM, by following a general procedure for all the soluble and immobilized enzymes. The enzyme preparation of PGA (15 U_PGK_) was added to the substrate solution at 4°C, under stirring (200 rpm). During the reaction, the pH was kept constant by automatic titration. The vs/vh_1 _ratio was evaluated by measuring the initial rate of the synthesis (vs) and acid formation (vh_1_) before the 20% of the ester was consumed. The reactions were monitored by HPLC analysis by using a device that consists of a KONTRON 422 pump, of a 3.9 × 300 mm C18 (INTERCHROM) reverse-phase column and of a 535 KONTRON detector with registration at 220 nm. The analytical conditions were 30% acetonitrile in 10 mM phosphate buffer, pH 3.1, flow 1.0 ml/min.

## Authors' contributions

DC carried out most of mutagenesis, expression, purification, immobilization and assay experiments and wrote the draft. IS carried out some of the HPLC PGA assays. DU wrote the final version of the paper and helped DC and IS in the organization of the experiments for immobilization and enzymes activity assays. AMA planned and did some of the experiments for mutagenesis, expression and purification and wrote the draft. MT with AMA conceived the study and organized the funding. All authors read, reviewed and approved the final manuscript.
